# The Role of the Gut Microbiome in Health and Disease in the Elderly

**DOI:** 10.1007/s11894-024-00932-w

**Published:** 2024-04-20

**Authors:** Lea Ann Chen, Kaitlyn Boyle

**Affiliations:** 1 Division of Gastroenterology and Hepatology, Department of Medicine, Rutgers, New Brunswick, NJ USA; 2 Rutgers Robert Wood Johnson Medical School, New Brunswick, NJ USA

**Keywords:** Microbiota, Aging, Geriatric, Probiotics, Frailness, Aged, 80 and older

## Abstract

**Purpose of Review:**

Growing evidence supports the contribution of age in the composition and function of the gut microbiome, with specific findings associated with health in old age and longevity.

**Recent Findings:**

Current studies have associated certain microbiota, such as *Butyricimonas*, *Akkermansia*, and *Odoribacter,* with healthy aging and the ability to survive into extreme old age. Furthermore, emerging clinical and pre-clinical research have shown promising mechanisms for restoring a healthy microbiome in elderly populations through various interventions such as fecal microbiota transplant (FMT), dietary interventions, and exercise programs.

**Summary:**

Despite several conceptually exciting interventional studies, the field of microbiome research in the elderly remains limited. Specifically, large longitudinal studies are needed to better understand causative relationships between the microbiome and healthy aging. Additionally, individualized approaches to microbiome interventions based on patients’ co-morbidities and the underlying functional capacity of their microbiomes are needed to achieve optimal results.

## Introduction

Over the past several decades, scientists have established an extensive relationship between the gastrointestinal (GI) microbiome and host health. For example, commensal microbiota contribute to host immune system development and function [1], with disruptions potentially contributing to immune-mediated diseases such as systemic lupus erythematosus (SLE) and inflammatory bowel disease (IBD) [2, 3]. Microbiome composition and function further influence the metabolism of nutrients and drugs [4, 5]. Growing research also suggests an important role for microbes in the gut-brain axis that modulates neuropsychological and sensory disorders, such as autism and irritable bowel syndrome (IBS) [6, 7].

Scientists have yet to identify one specific healthy microbiome, and it is generally agreed upon that there is no singular “normal” composition [8]. This is likely due to the vast spectrum of factors that influence the gut microbiome, including variations in diet [9], genetics [10], and environment [11]. As such, changes in the microbiome also occur as part of the natural aging process. Microbiome development begins at birth as soon as newborns exit the vaginal canal and encounter the mother’s vaginal fluids [12], or alternatively through the skin and the environment for babies delivered by Cesarean section [13]. For breast-fed infants, breastmilk contains several prebiotics (e.g., human milk oligosaccharides) which selectively support the growth of beneficial bacteria in the GI tract [13]. As children transition to solid foods, they encounter new dietary components such as starches and cell wall polysaccharides; their microbiomes must then shift to select for bacteria capable of metabolizing these nutrients [14]. While changes in infant and early childhood microbiomes have been studied extensively, there is less information regarding alterations in the gut microbiome of the elderly. Accordingly, there is growing interest in understanding the effect of aging on the host microbiome and whether aging and its associated features, such as frailty and declined cognition, can be modulated by gut bacteria. According to the United Nations, the number of people over the age of 65 worldwide in 2021 was 761 million, with that number expected to rise to 1.6 billion by 2050 [15]. A greater understanding of this population’s microbiome is thus of growing relevance in addressing human health and disease.

Increasing research suggests that natural, or “healthy”, aging leads to specific changes in gut microbiome composition, such as the loss of certain commensal genera, including *Prevotella*, *Faecalibacterium*, and *Bifidobacterium*, and the species *Eubacterium rectale* [16, 17]. These taxa are instead replaced at older age by other commensal organisms, such as *Butyricimonas*, *Akkermansia*, and *Odoribacter* [12, 16–18]. *Akkermansia muciniphila*, in particular, has been widely studied in aging and disease and is known to contribute to mucin degradation in the intestines [19]. Some have speculated that *Akkermansia* levels can indicate health status, with an increased relative abundance (above that seen in healthy aging) associated with excellent health among centenarians and a decreased relative abundance associated with thinning of the gut mucus layer and decreased acylglycerol, [18, 20] an endocannabinoid that regulates gut permeability and decreases intestinal inflammation [18, 20].

Scientists have also identified several pathobionts, or conditionally pathogenic microorganisms, that are increased in “unhealthy” aging [17, 18], a process characterized by rapid physical and mental decline and associated with disease progression and physical frailty. Some of these pathobionts include *Eggerthella*, *Actinomyces*, and *Enterobacteriaceae*, the presence and quantity of which may help physicians predict lifespan and disease outcomes [17, 18].

One challenge in conducting and interpreting microbiome studies in the elderly is distinguishing results attributable solely to age from those due to different states of health. Unique subjects by which to study these questions are those who are of “extreme” old age, such as centenarians (≥ 100 years old) and supercentenarians (≥ 110 years old). Microbiome features of these extremely aged individuals presumably confer longevity rather than any deleterious aspects of aging. For example, bacterial strains that are often decreased in the elderly, such as *Christensenella* and *Bifidobacterium*, are actually increased in semi-supercentenarians (i.e., 105–109 years old) [18], suggesting their beneficial effect. Additionally, the highly studied *Akkermansia* taxon, which is abundant in healthy aging, is even more dramatically increased in extreme aging [18].

To further clarify the independent contributions of age and health, Wilmanski et al. cross-sectionally evaluated gut microbiome compositions by decade of life in 3653 U.S. adults, aged 18–87 years old. The authors found that starting around 40–60 years old, individuals become more “unique” in their microbiomes as measured by the Bray–Curtis dissimilarity matrix, in which individuals’ microbiomes were compared to the one most similar from the remaining subjects [21••]. More microbiome dissimilarity was seen with each passing decade, a finding that was consistent regardless of sex, body mass index (BMI), and alpha diversity (i.e., within-sample diversity). Samples in this analysis were evaluated in two separate groups (*n* = 2539 and *n* = 1114) as there was a change in microbiome vendors and sample processing during the study; nevertheless, the finding of greater microbiome dissimilarity with increasing age was present in each cohort, lending further strength to the results. In the same publication, the investigators analyzed a different cohort of only men, aged > 78 yo (*n* = 599 discovery cohort and n = 308 validation cohort). Again, microbiome uniqueness, as measured by Bray–Curtis dissimilarity, was positively correlated with age. Notably, the strength of this correlation increased among healthy participants, as determined by medication use, self-perceived health, life-space score (LSC) [22], and walking speed. When sub-analyzing the community dwellers of this male cohort (i.e., those who did not reside in nursing homes, assisted living, or were hospitalized in the past 12 months; *n* = 706), investigators identified a correlation between the relative abundance of *Bacteroides* and all-cause mortality, independent of multiple potential confounders (e.g., age, BMI, and self-perceived health). This association between *Bacteroides* abundance and mortality was even stronger among community dwelling subjects in this cohort 85 + years old, suggesting the potential use of decreased *Bacteroides* as a longevity biomarker [21••]. In contrast, a large 2023 cohort study (*n* = 1575, including 297 centenarians) found a relative abundance of *Bacteroides* in centenarians and furthermore identified several other “youth-associated” features, such as decreased pathobionts, in centenarians [23]. Thus, the role of *Bacteroides spp.* and their utility as a gut microbial predictor of long life requires additional study. Another growing area of research is exploring the centenarian virome, with early results suggesting increased viral diversity and unique genera enrichment that distinguish centenarians from other older adults (> 60) [24•].

The logical next steps in this line of research will be longitudinal studies of the microbiome to determine whether microbes associated with longevity are present earlier in life (suggesting their role in predicting or promoting long life) or if their increase occurs only upon old age, suggesting that these changes are relevant only in older age or are secondary to other factors of extreme age.

## Geriatric Health and the Microbiome

**Fig. 1:**
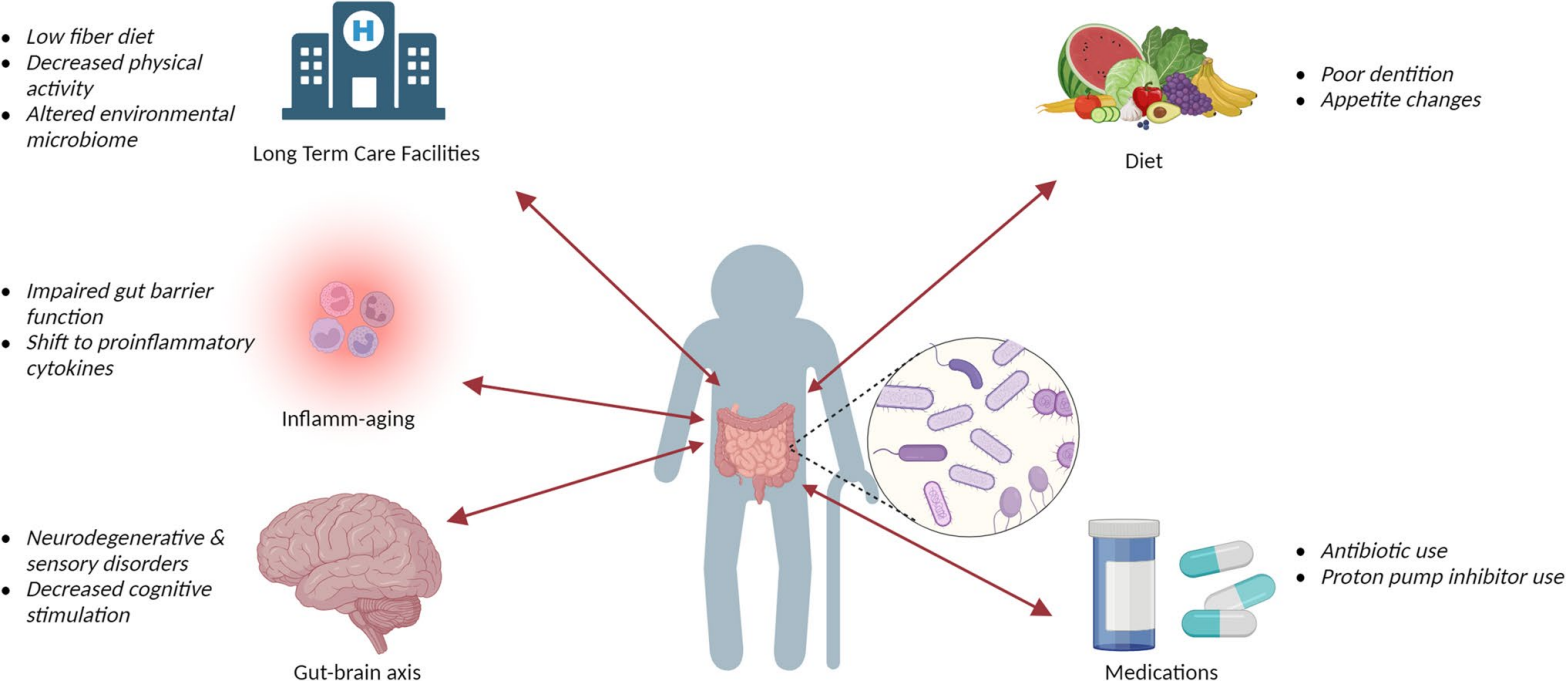
Lifestyle changes and medical conditions associated with alterations in the intestinal microbiome of the elderly

Although we know that microbiome compositions shift throughout the aging process, the exact mechanisms for this are unclear. Below, we explore some common life changes and medical conditions among the elderly in which intestinal microbiomes are altered (Fig. 1).

### Inflamm-aging

Studies from as early as the 1960’s have indicated a decrease in immune function in aging adults [25]. This process, now known as immunosenescence, is associated with a decline in immune system function that leads to an accumulation of pro-inflammatory cytokines. The increased inflammatory state in elderly populations is now commonly referred to as “inflamm-aging” [26]. Pro-inflammatory states place patients at higher risk for a variety of conditions such as autoimmune and cardiovascular diseases, as well as infections [27–29].

Within the GI tract, the maintenance of functioning epithelial and mucus barriers is essential for protection against infection and disease [30]. Increased intestinal permeability can lead to translocation of microbes into host circulation, exacerbating a pro-inflammatory state [30]. A study in wildtype C57BL/6 mice showed that age-associated disruption of the small intestine mucosal barrier led to increased interaction between gut microbiota and the host immune system, as determined by fluorescent in situ hybridization using the bacterial probe EUB338-Alexa Fluor 488, as well as enlargement of solitary intestinal lymphoid tissue (SILT), which are hypertrophied upon interaction with gut microbes [31, 32]. Barrier defects were also associated with relative decreases in *Akkermansia* [31].

Experiments in germ-free mouse models by Thevaranjan et al. suggest that it is the changing microbiome itself in aging populations that leads to a pro-inflammatory state, with germ-free mice living much longer than their conventional counterparts [33]. Furthermore, young, germ-free mice gavaged with the microbiome of older mice developed greater intestinal permeability and circulating TNF than mice gavaged with microbiomes of other young mice [33]. However, additional research, especially from longitudinal studies in humans, will be necessary to confirm a causal relationship between the gut microbiome and inflamm-aging.

### Diet and Environment

Elderly individuals requiring greater assistance with activities of daily living (ADLs) may transition from community living to long-term care facilities. This relocation has been shown to produce microbiome shifts due to presumed changes in environmental, dietary, and medical factors [34]. For instance, in general adult population studies, the microbes residing on household surfaces correlate with gut microbiome composition, which bears consideration in the transition to long-term care environments. Furthermore, both aging and exposure to healthcare facilities, such as long-term care facilities, are associated with an increased risk for *Clostridioides difficile* infection (CDI), a major cause of healthcare-associated, inflammatory diarrhea [35].

Regardless of age, there is strong evidence to suggest that specific diets can cause unique alterations in the microbiome [36, 37], as well as corresponding serum and fecal metabolites [38]. A well-controlled study by Tanes et al. followed 30 subjects who were randomized to vegan (high fiber), omnivore (intermediate fiber) and formula-based (no fiber) diets [39]. After 6 days, the subjects were given a “gut purge” using a combination of oral antibiotics and polyethylene glycol. Researchers found that the microbiome of vegan subjects recovered more rapidly after the “purge” compared to the other groups, regaining greater diversity in a shorter time span [39]. Subjects adhering to the formula-based diet, on the other hand, had the most prolonged recovery phase. In another cross-sectional study, the gut microbiomes of previously uncontacted Yanomami Amerindians, who live in the High Orinoco state of Venezuela and eat a largely plant-based, high-fiber diet, were compared to microbiomes of individuals residing in the United States and semi-transculturated populations, such as Guahibo Amerindians and Malawians. The Yanomami were noted to have a markedly higher gut microbiome diversity compared to those in the United States, with the semi-transculturated populations having an intermediate level of diversity. It is noted, however, that other social and medical factors, rather than diet alone, could also have contributed to this increased diversity [40].

Nevertheless, a component of age-related changes in the microbiome appears definitively related to diet and eating, particularly as the elderly are at increased risk for poor dentition or chewing difficulties, decreased appetite, and lack of social support in obtaining nutritious foods [41]. For example, one of the most dramatic diet changes that has been shown to cause microbiome alterations is the move from independent, community living to assisted living within a long-term care facility. This transition often leads to a change from a high-fiber, low-fat diet to a low-fiber, high-fat diet, which has been associated with a shift to a lower diversity microbiome in long-term care residents compared to community dwelling counterparts [34]. Of note, the disparity between these long-term care residents and community dwellers correlated with the amount of time spent in long-term care. During digestion, fiber is metabolized into short chain fatty acids (SCFAs), which provide many benefits to the GI tract by serving as an energy source for protective microbiota, assisting with anti-inflammatory responses, and maintaining gut barrier integrity [42]. Thus, SCFA deficiencies caused by dietary changes when moving to long term care facility can indirectly contribute to intestinal dysfunction.

### Co-morbidities

An area of growing interest is the study of the gut-brain axis via microbes that may influence cognitive function (Table 1). The topic is of particular relevance as mild cognitive impairment (MCI) is highly prevalent in the elderly, affecting approximately 10% of those aged 70–74 yo and 25% of those 80–84 yo [43]. Furthermore, patients with MCI are far more likely to progress to dementia [43]. Pharmacologic treatments to date can only slow the progression of MCI, but not reverse it [44]. While there is still disagreement on whether microbiome alterations influence cognitive function and vice versa [45, 46], ongoing long-term projects such as MOTION (Microbiome Of the ageing gut and its effect on human gut health and cogniTION), which studies cognitive and microbiome changes of healthy aging [47], provide hope that these interactions will soon be clarified.

**Table 1:** Selected Cognitive Function Studies. Select randomized control trials and observational studies (2019–2023) that evaluate cognitive function and the gut microbiome in the elderly

Reference	Study Design	Species	Intervention	Sample Size	Duration	Outcome
[48••]	RCT	Human	Mediterranean Diet counseling	612 (289 control, 323 experimental)	1 year	Increased taxa associated with cognitive function, non-frailty, and reduced inflammation
[49]	RCT	Human	*Bifidobacterium bifidum* BGN4 and *Bifidobacterium longum* BORI probiotic	53 (26 placebo, 27 probiotics)	12 weeks	Probiotic group with improved mental flexibility and brain-derived neurotrophic factor and decreased inflammation-associated taxa
[50]	RCT	Human	*Lactobacillus plantarum* C29-fermented soybean (DW2009) supplement	100 (50 placebo, 50 probiotic)	12 weeks	Improved cognitive function, increased brain-derived neurotrophic factor
[51]	RCT	Human	*Bifidobacterium breve* MCC1274 probiotic	115 suspected MCI patients (60 placebo, 55 probiotic)	24 weeks	Improved cognitive function in MCI patients
[52]	Cross-sectional observational	Human	N/A	69	N/A	Association between certain taxa and specific domains of cognition (e.g. *Alcaligenaceae* and *Clostridiaceae* and working memory)
[53]	Cross-sectional observational	Human	N/A	48 (22 MCI, 26 healthy control)	N/A	Altered microbiome profiles between MCI and healthy controls
[54]	Cross-sectional observational	Human	N/A	127 (75 MCI, 52 healthy control)	N/A	MCI patients showed decreased microbial diversity, relative abundance of *Faecalibacterium*, Ruminococcaceae, and *Alistipes*; elevated *Proteobacteria* and *Gammaproteobacteria*
[55]	Cross-sectional observational	Human	N/A	90 (30 AD, 30 MCI, 30 healthy control)	N/A	Altered microbiome features seen in AD patients present earlier than symptom development (i.e. in MCI patients)
[56]	Observational	Human	FMT for CDI	20 dementia patients with *Clostridium difficile* infection (10 FMT recipients, 10 antibiotics only recipients)	N/A	FMT improved cognitive function in dementia patients
[57]	Observational	Human	N/A	97 (33 AD, 32 MCI, 32 healthy control)	N/A	Reduced alpha diversity and altered microbiome composition in AD compared to MCI patients and to healthy controls
[58]	Cross-sectional/ RCT	Human/ Mouse	Human: N/A Mouse: *Faecalibacterium prausnitzii* gavage	Human: 41 (21 healthy, 15 MCI, 7 AD) Mouse: 108 (9 mice to each *F. prausnitzii* isolate)	Human: N/A Mouse: 11 days	Human: MCI patients had decreased *F. prausnitzii* Mouse: *F. prausnitzii* administration in AD mouse model improved cognitive function
[59]	RCT	Mouse	FMT	22 recipients (11 treated with pooled aged donor stool, 11 treated with pooled young donor stool)	90 days	Recipients of aged FMT showed impaired memory function
[60]	RCT	Mouse (senescence-accelerated mouse prone 8 (SAMP8))	Probiotic composed of *Bifidobacterium lactis*, *Lactobacillus casei*, *Bifidobacterium bifidum*, and *Lactobacillus acidophilus*	24 (12 water, 12 probiotic mixture)	12 weeks	Improved memory deficits, intestinal barrier dysfunction, and blood–brain barrier dysfunction

A 2019 study of shotgun metagenomic sequences, comparing 57 nursing home residents with dementia, including Alzheimer’s disease (AD), with 51 elderly individuals without AD or other forms of dementia, revealed higher levels of pro-inflammatory gut bacteria in those with dementia [61]. The authors also noted a decrease in butyrate-synthesizing bacterial species, such as those in the genera *Butyrivibrio* and *Eubacteria,* in the AD group when compared to both subjects without dementia and subjects with other dementias besides AD [61]. A subsequent systematic review and metanalysis similarly found decreased alpha diversity in the gut microbiomes of AD patients compared to healthy controls, but not between those with mild cognitive impairment (MCI) and healthy controls. Differences in microbiome compositions between AD, MCI, and healthy samples (i.e., beta diversity) were not consistently altered [62]. One challenge in studying the gut microbiome as it relates to dementia is the lack of clear, objective, and non-invasive tests to conclusively determine diagnosis and disease stage, thus further complicating the interpretation of study results. While beyond the scope of the gut microbiome, we note with interest that post-mortem studies of AD brain tissue have identified the presence of microbes within the brain, suggesting the presence of a brain microbiome associated with neurodegenerative disease [63].

Furthermore, a large genome wide association study identified several microbiome genera associated with high risk alleles of the apolipoprotein E ε4 (APOE ε4) gene, a well-established risk factor for AD [64•]. Some of the most significant findings of this study included a strong correlation between the pro-inflammatory genus *Collinsella* and APOE risk alleles, as well as a proposed protective role for the genus *Eubacterium fissicatena* [64•].

Parkinson’s disease (PD) is another neurological disorder that is more common in the elderly and for which there is growing interest in the gut microbiome as a biomarker or therapy. A 2020 meta-analysis of 16S sequencing data from Japan, the United States, Finland, Russia, and Germany found that patients with PD have relatively decreased *Roseburia* and *Faecalibacterium –* both important producers of the SCFA butyrate [65]. A 2022 shotgun sequencing study of 490 PD and 234 healthy controls confirmed these findings and also identified several other genera that are altered in PD patients, such as an increase in pathogenic species of *Prevotella* [66•]. Interestingly, multiple studies have noted an increase in the *Akkermansia* genus [65] among those with PD. This is surprising considering *Akkermansia* is generally associated with healthy aging and is particularly abundant in supercentenarians [18]. Some scientists have speculated that *Akkermansia* is an important component of healthy aging, but that increased abundance puts patients at risk for neurocognitive disease [67]. We further hypothesize that changes in *Akkermansia* abundance may be secondary to the development of constipation, a common gastrointestinal complication of PD and a condition that has independently been associated with increased *Akkermansia* in multiple other studies [68]. As the link between PD and *Akkermansia* is an inconsistent finding [66•], further research is needed to determine the precise role of this genus in PD and in aging more broadly. In a PD mouse model that overexpresses α-synuclein aggregates, a common finding in the brains of PD patients, mice colonized with the gut microbiome of 6 human PD patients had increased physical motor impairments and constipation compared to mice colonized with healthy donor microbiota [69]. Building on these early findings of altered microbiota in PD, a pilot randomized control trial found that stool from healthy donors, given as lyophilized pills twice a week for 12 weeks, could improve constipation and gut motility as well as transiently improve objective motor skills among patients with mild to moderate PD [70•]. While significant translational and clinical data development are still needed, these initial findings maintain the promise that gut microbiome modulation may improve gastrointestinal and/or neurological symptoms of PD and provide deeper insight into disease pathophysiology.

Several early-stage studies have also been conducted on the relationship between the gut microbiome and sarcopenia, the progressive deterioration of muscle mass that occurs with aging and that leads to physical frailty. While these studies have yielded conflicting results about which bacterial species are increased or decreased in the condition, study findings have consistently demonstrated no change in overall microbial diversity between frail and non-frail elderly individuals [71, 72]. Pre-clinical experiments have also suggested a role for gut bacteria in skeletal health, although details of how these effects are mediated have been unclear [73, 74]. In correlating human subject research, a relatively large 16S study (i.e., 60 individuals with osteoporosis and 60 age- and gender-matched controls with normal bone mineral density) found a relative abundance of *Actinomyces, Clostridium XIVa, Eggerthella,* and *Lactobacillus* and a relative decrease in *Veillonella* in those with osteoporosis. There were, however, no changes in overall microbiome alpha diversity between groups [75].

## Interventions to Delay or Reverse Aging

While a more thorough understanding is required of the microbial changes that can be isolated to age specifically, studies have already begun evaluating how to restore a healthy microbiome in aging populations to promote health and longevity.

### FMT

Fecal microbiota transplant (FMT) is a therapy that has been growingly incorporated into the treatment of recurrent CDI [76] and has furthermore been studied for inflammatory bowel disease [77] and post-antibiotic dysbiosis [78]. During FMT, stool from a healthy donor is transplanted into a recipient via colonoscopy, naso- or oro-enteric tubes, enema, or capsule, with the goal of transferring the corresponding intestinal microbes, as well as their contained functions and metabolic products. This has led some to speculate whether the microbiome from a young, healthy donor can be transplanted into an elderly individual to reverse some of the effects of unhealthy aging (Table 2).

**Table 2:** Selected FMT Findings. Select randomized control trials (2019–2023) that investigate fecal microbiota transplant (FMT) in the aging microbiome

Reference	FMT Donor	Donor Characteristics	FMT Recipient	Duration	Microbiome Findings	Physiological Findings
[79•]	Mouse	C57BL/6 J mice aged 3, 18, or 24 months	C57BL/6 J mice aged 3, 18, or 24 months	18 days	Aged FMT donor: increased *Prevotella*, *Lacnospiraceae*, *Facecalibaculum* Young FMT donor: increased *Bifidobacterium animali*s, *Akkermansia muciniphil*a	Aged FMT donor: increased CNS inflammation, cognitive decline, loss of intestinal epithelial barrier Young FMT donor: increased vitamin and lipid synthesis, decreased inflammation
[59]	Mouse	WT young (2–3 months) or aged (18–20 months) mice	C57BL/6 GF mice (12 weeks)	90 days	Aged FMT donor: decrease in SCFA-producing bacteria	Aged FMT donor: decrease in memory and cognition, increase in depressive behavior
[80]	Mouse	C57BL/6 J mice (aged 24 months or young 3 months)	C57BL/6 J mice (3 months)	35 days	Aged FMT donor: decrease in SCFA-producing bacteria, *Prevotellaceae*, *Ruminococcaceae*	Aged FMT donor: decreased memory and learning, altered expression of hippocampus proteins
[81]	Mouse	WT mice or advanced-age (i.e., 4 months) LmnaG609G/G609G progeria mice	LmnaG609G/G609G mice (8–10 weeks)	Until deceased (Max 289 days)	N/A	Progeroid mice receiving WT FMT had increased survival and decreased intestinal inflammation cytokines. Progeroid mice receiving advanced age progeroid FMT had reduced lifespans
[67]	Mouse	Young SAMP8* (2–3 months) or aged SAMP8 (10–11 months) mice	SAMP8 mice (7 months)	6 months	Aged FMT donor: increased *Akkermansia*	Young FMT donor: increased locomotion, exploration
[82]	Mouse	Young mice (5 weeks) stool or no FMT	Aged mice (42 weeks)	10 weeks	Increased *Bifidobacterium* and *Ruminococcaceae*, decreased inflammatory markers	Decreased ovarian atresia and ovarian follicle apoptosis, and increased first litter size in FMT-treated female mice
[83]	Mouse	Young C57BL/6 mice (5 weeks)	Aged C57BL/6 Mice (12 and 25 months)	8 weeks	Increased *Muribaculaceae*, *Bacteroidaceae*, and *Prevotellaceae*	Increased muscle function/size, skin integrity
[84]	Mouse	5xFAD Alzheimer’s disease model mice	WT mice young (4 weeks) or aged (12 months)	6 weeks	Aged FMT donor: increased *Lactobacillaceae* in both young and aged mice	Aged FMT donor: increased plaque deposition in brain in both young and aged mice
[85]	Mouse	Young C57BL/6 mice (3 months)	Aged C57BL/6 mice (22 months)	23 days	N/A	FMT Improved germinal center reactions in Peyer's patches from donor of different age
[86]	Mouse	C57BL/6 mice young (7–8 weeks) or aged (20–24 months)	Aged C57BL/6 mice (20–24 months)	4 weeks	Increased *Butyricicoccu*s, Lachnospiraceae, *Clostridium*; decreased *Bacteroides*, *Alistipes* and *Anaeroplasma* in recipients of young-donor FMT compared to aged-donor FMT	Increased lymphoid function, hematopoietic stem cell function capacity, intestinal barrier function in recipients of young-donor FMT compared to aged-donor FMT
[87]	Human	Long living (101 year) or aged (70 year) human subject stool	C57BL/6 J mice (11 months)	12 weeks	Long-living donor: increased α diversity and SCFA** producing genera	Long-living donor: increased intestinal villi length
[88]	Zebrafish	Young zebrafish (4 months)	Aged zebrafish (3 years)	4 weeks	N/A	Protective effect against toxic pollutant, increased reproductive function

Footnotes:

*SAMP8 = Senescence Accelerated Mouse-Prone 8

**SCFA = Short Chain Fatty Acid

A study by Parker et. al demonstrated that transfer of an “aged” microbiome from elderly mice to younger mice caused several age-associated phenotypes including advanced central nervous system deterioration and vision deficits [79•]. Importantly, in a set of correlating experiments, age-related changes improved in elderly mice after microbiome transplantation with stool of younger mice [79•]. This work provides strong pre-clinical evidence that microbiome profiles between young and aged mice are not only different, but that the associated physiological effects of these microbiomes are transferrable. These and similar findings have been reproduced by other investigators [59], including D’Amato et al., who demonstrated that transferring the microbiome of elderly mice to young ones can lead to cognitive deficits [80].

Progeria is a particularly unique disease by which to study microbiome and senescence, as affected individuals carry a mutation in the gene encoding lamin A which leads to rapid aging. Despite a normal appearance at birth, affected individuals typically develop fatal complications of their disease, predominantly cardiovascular disease, in their teens or early adulthood [89]. Using a mouse model of progeria, Bárcena et al. showed that certain bacterial strains enriched in human centenarians such as *Akkermansia muciniphila* can be transplanted to increase mouse lifespan and to reverse intestinal mucosal thinning [81]. Although these findings are still in the preclinical phase, they hold exciting promise for the use of FMT from young donors, or its therapeutic components, to reverse certain aspects of unhealthy aging.

### Diet and Probiotics

As discussed previously, elderly populations often have variations in diet as they age, which contribute to microbiome changes. One of the most studied dietary changes associated with aging is reduced fiber intake; however clinical trials supplementing fiber have yielded conflicting results regarding shifts in microbiota composition and inflammatory status [90, 91], with some researchers hypothesizing that the efficacy of dietary interventions and supplements may depend on the host’s initial microbiome profile. For example, in a double-blind, crossover trial of 21 healthy volunteers over 60 years old who were given supplemental wheat bran-derived arabinoxylan-oligosaccharide found that resulting microbiome compositions varied based on subjects’ initial *Prevotella* abundance [92]. Although limited, these findings suggest that an individualized approach is required to manipulate the microbiome, with screening of patients’ initial microbiomes necessary to tailor the intervention needed for the desired outcome.

In addition to specific supplements, certain diets have been associated with gut health. The Mediterranean diet, consisting of plant-based foods, whole grains, and healthy fats, has been shown to prevent cardiovascular disease in all age ranges [93], with the effects of this diet potentially mediated by the gut microbiome. For example, a 2020 study by Ghosh et al. found that adherence to the Mediterranean diet for at least one year corresponded to a relative increase in intestinal *F. prausnitzii*, *R. hominis*, *E. rectale*, *E. eligens*, *E. xylanophilum*, *B. thetaiotaomicron*, *P. copri* and *A. hadrus* [48••]*.* Adherence to the diet furthermore correlated with improved cognitive function, as measured by the BabCock Memory Score and Constructional Praxis, as well as decreased systemic inflammatory markers such as high-sensitivity C reactive protein (hsCRP) and interleukin 17 (IL-17) levels. Mouse studies have also demonstrated that a Western diet, which is high in fat and sodium, leads to an increased “predicted age” of the gut microbiome based on a Bayesian model trained on male C57BL/6 J mice whose microbiomes were characterized from week 9 to week 112 of life. These microbiome disturbances reversed once the mice returned to a standard diet [94]. Interventional diet studies evaluating both the gut microbiota and clinical outcomes in elderly, human cohorts are therefore of particular interest given these individuals’ susceptibility to cognitive decline and unhealthy aging.

Probiotic interventions have been specifically studied in the aged. Unfortunately, similar to studies in the general population, the generation of clinically actionable data has been dampened by the great heterogeneity of studied products and outcomes as well as the multitude of underpowered studies [95]. While no singular or combination of probiotic organisms have been identified to definitively improve or reverse signs of aging [96], a growing number of studies are evaluating specific microbial strains and their impact on objective physiological effects. For example, in a double blind, placebo controlled study, *L. reuteri* ATCC PTA 6475 supplementation in elderly women with low bone mineral density improved tibia total volumetric BMD (vBMD) [97, 98]. Furthermore, in the Senescence Accelerated Mouse-Prone 8 (SAMP8) mouse model, probiotic *Lactobacillus casei Shirota* administration reduced age-related muscle deterioration and mitochondrial dysfunction [99]. In humans, some small, but double-blinded randomized controlled trials have identified specific probiotics that appear to improve cognitive function in elderly adults, especially probiotics that include *Bifidobacterium* and *Lactobacillus spp.* [49, 50]. Thus, as a more rigorous understanding between microbiome manipulation and objective health measures develops, probiotic therapies may entail customized cocktails of microorganisms to target specific deficiencies or conditions in a personalized approach to care.

### Exercise

Multiple studies have reported an alteration in the gut microbiome following the implementation of an exercise program [100], with early results suggesting that this is true in elderly populations as well [101–103]. A 2020 study by Zhu et al. utilized fecal specimens from the American Gut Project, which also included patient-reported information on BMI and exercise habits [102]. The study included samples from 1,589 adults (aged 18–60 years) with a healthy BMI (18.5 ≤ BMI ≤ 25) and 897 elderly patients (aged > 60), who were further stratified by BMI into normal weight (*n* = 462), overweight (BMI > 25, *n* = 413) and underweight (BMI < 18.5, *n* = 22), as well as by exercise frequency. Investigators found that as the reported frequency of exercise increased in elderly patients, the microbiome of the elderly patients more closely resembled that of the healthy BMI adults based on the relative abundance of specific taxa and common pathways. For example, the relative abundance of *Actinobacteria* in exercising elderly adults increased compared to non-exercising elderly adults and approached the levels seen in healthy BMI adults. Furthermore, the relative abundance of *Cyanobacteria*, decreased in exercising elderly patients, again approaching levels seen in healthy BMI adults. (Of note, however, *Cyanobacteria* produce toxins such as β-N-Methylamino-l-alanine (BMAA) have been implicated in neurodegenerative diseases such as AD and ALS [104, 105].) In a smaller study by Erlandson et al., 15 sedentary elderly patients (aged 50–75) were recruited for a supervised 24 week, thrice-weekly cardiovascular and resistance exercise program. Stool samples were collected before and after the intervention for 16S sequencing [103]. Researchers observed an increased relative abundance of *Bifidobacterium* after 24 weeks of the exercise program, as well as increased butyrate levels. Considering the speculated role of *Bifidobacterium* in extreme aging and improved cognitive function, these findings suggest that the health benefits related to exercise may also be mediated through the gut microbiome.

Despite these results, there is significant interpersonal variation in the reported microbiome changes that occur with exercise [106]. Additionally, many of the current studies do not have a control arm, lack rigor, and/or have small sample sizes. Future studies are needed to identify if there is in fact a relationship between exercise and healthy aging microbiota, as well as the type of physical activity that can influence gut microbiomes.

## Conclusions

Microbiome research in the elderly is an exciting, rapidly growing field; however, a major gap in the literature is the lack of longitudinal data by which to distinguish between causative and correlative relationships given the many concomitant changes that occur with age, including altered dentition, diet, sleep, and lifestyle patterns. A challenge in interpreting currently available data is the difference in sequencing methodologies utilized. For instance, 16S study results can differ depending on the portion of the variable region within the 16S gene that is being sequenced [107], as well as due to variation in 16S copy number between bacterial species [107, 108]. Comparing 16S data across studies is also challenging as the results provide only the relative abundances of taxa in a group [109]; thus, abundances of one group may appear to change but only because of changes in the abundance of other taxa [110]. Furthermore, as taxonomy does not necessarily inform microbial function [111], it is likely that a future shift in focus to metagenomic function may better clarify the mechanisms by which gut microbes influence their host.

Additional challenges specific to clinical microbiome studies in the elderly and the extremely elderly include difficulties with mobility, the tendency for increased medical co-morbidities, as well as difficulties determining capacity for decision-making, and finding a proxy for subjects who may not be able to provide their own consent to participate in research.

Nonetheless, early findings suggest that there is the potential to reverse microbiome aging with interventions such as FMT, exercise, and dietary modifications. Several large, longitudinal cohort studies are now underway that aim to characterize microbiome changes throughout aging, including the MOTION study and a new branch of the Wisconsin Longitudinal study (WLS) [47, 112]. As mentioned previously, the MOTION study is a longitudinal, prospective cohort study of 360 healthy individuals over the age of 60, that is specifically interested in the relationship between cognitive function and gut microbiome in elderly populations [47]. The WLS, a longitudinal study of one-third of the Wisconsin high-school graduates in 1957, recently incorporated a microbiota branch to the project through the collection of 429 stool specimens (74% response rate), which will analyze the gut microbiome as it relates to environmental conditions and disease development [112].

As the field of medicine becomes more individualized with the growth of genetics, epigenetics, and other biomarkers, we must also consider the importance of a unique microbiome profile in diagnosis and treatment. A common theme throughout much of the research is the significance of individualized care, with treatments based on the initial host microbiome composition. Although many of these studies are still in the early stages and require additional evidence to confirm a true causative relationship between illness and dysbiosis, elucidation of a unique microbiome disease profile opens the door to new avenues of treatment for these diseases.

## Data Availability

No datasets were generated or analysed during the current study.
